# 16α,17α-Ep­oxy-5α-hydr­oxy-6β-nitrooxy-20-oxopregnan-3β-yl acetate

**DOI:** 10.1107/S1600536809016997

**Published:** 2009-05-14

**Authors:** R. M. A. Pinto, A. Matos Beja, J. A. R. Salvador, J. A. Paixão

**Affiliations:** a Laboratório de Química Farmacêutica, Faculdade de Farmácia, Universidade de Coimbra, P-3000-295 Coimbra, Portugal; b CEMDRX, Departamento de Física, Faculdade de Ciências e Tecnologia, Universidade de Coimbra, P-3004-516 Coimbra, Portugal

## Abstract

The title steroid, C_23_H_33_NO_8_, is a pregnane derivative obtained regio-, stereo- and chemoselectively from the ring opening of the corresponding 5α,6α;16α,17α-diepoxide with bis­muth(III) nitrate. There are two symmetry-independent mol­ecules in the asymmetric unit that show no significant differences concerning bond lengths and angles. All rings are *trans*-fused. The conformations of the six-membered rings are close to chair forms, while the five-membered ring adopts an envelope conformation. The mol­ecules are held together by an extensive O—H⋯O hydrogen-bonding network of chains runnning along the *a* axis.

## Related literature

For epoxy­steroid chemistry, see: Salvador *et al.* (2006 ▶, 2008 ▶); Pinto *et al.* (2008*a* ▶). For the synthesis of β-hydr­oxy nitrates, see: Pinto *et al.* (2007*a* ▶). For the structures of 5α-hydr­oxy-6β-substituted steroids, see: Pinto *et al.* (2007*b* ▶, 2008*b* ▶,*c* ▶). For puckering parameters, see: Cremer & Pople (1975 ▶).
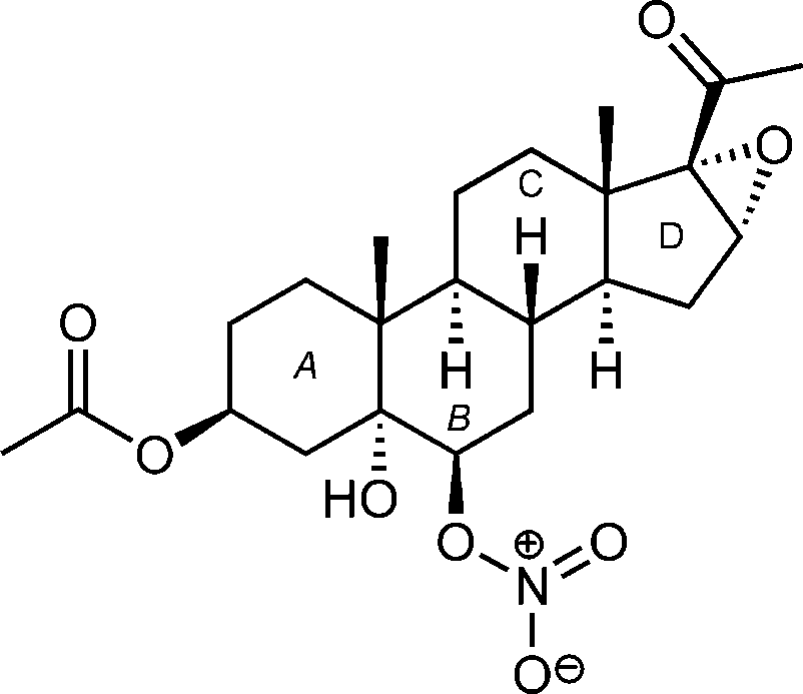


## Experimental

### 

#### Crystal data

C_23_H_33_NO_8_
*M* *_r_* = 451.50
Triclinic, 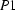

*a* = 10.9740 (3) Å
*b* = 11.0721 (3) Å
*c* = 11.1686 (3) Å
α = 77.2135 (15)°
β = 73.2087 (14)°
γ = 64.3465 (14)°
*V* = 1163.81 (6) Å^3^
*Z* = 2
Mo *K*α radiation
μ = 0.10 mm^−1^
*T* = 223 K
0.32 × 0.17 × 0.06 mm

#### Data collection

Bruker APEXII CCD area-detector diffractometer
Absorption correction: multi-scan (*SADABS*; Sheldrick, 2000 ▶) *T*_min_ = 0.947, *T*_max_ = 0.994
24934 measured reflections
5770 independent reflections
4806 reflections with *I* > 2σ(*I*)
*R*_int_ = 0.025

#### Refinement

*R*[*F* ^2^ > 2σ(*F* ^2^)] = 0.043
*wR*(*F* ^2^) = 0.118
*S* = 1.04
5770 reflections
587 parameters
3 restraints
H-atom parameters constrained
Δρ_max_ = 0.37 e Å^−3^
Δρ_min_ = −0.27 e Å^−3^

### 

Data collection: *SMART* (Bruker, 2003 ▶); cell refinement: *SAINT* (Bruker, 2003 ▶); data reduction: *SAINT*; program(s) used to solve structure: *SHELXS97* (Sheldrick, 2008 ▶); program(s) used to refine structure: *SHELXL97* (Sheldrick, 2008 ▶); molecular graphics: *PLATON* (Spek, 2009 ▶); software used to prepare material for publication: *SHELXL97*.

## Supplementary Material

Crystal structure: contains datablocks global, I. DOI: 10.1107/S1600536809016997/bt2943sup1.cif

Structure factors: contains datablocks I. DOI: 10.1107/S1600536809016997/bt2943Isup2.hkl

Additional supplementary materials:  crystallographic information; 3D view; checkCIF report

**Fig. 1.**
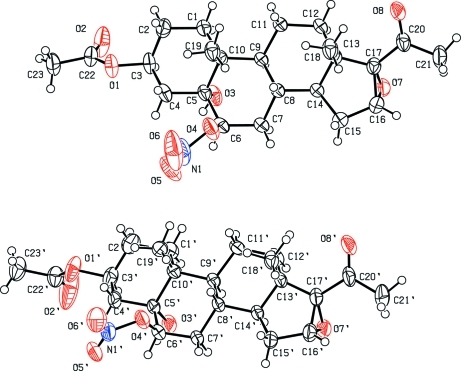
ORTEPII plot of the title compound. Displacement ellipsoids are drawn at the 50% level.

Enhanced figure: interactive version of Fig. 1

## Figures and Tables

**Table 1:** Hydrogen-bond geometry (Å, °)

*D*—H⋯*A*	*D*—H	H⋯*A*	*D*⋯*A*	*D*—H⋯*A*
O5*A*—H5*A*⋯O20*A*^i^	0.82	2.01	2.822 (2)	168
O5*B*—H5*B*⋯O20*B*^i^	0.82	2.11	2.913 (2)	168

Symmetry code: (i) 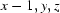.

## supplementary crystallographic information

### Comment

Epoxysteroids are amongst the most versatile intermediates in the synthesis of biologically important active molecules (Salvador *et al.*, 2006, 2008; Pinto *et al.*, 2008*a*). Recently, we have reported a new process for the preparation of β-hydroxy nitrates by the use of stoichiometric amounts of bismuth(III) nitrate, in the ring opening reaction of epoxides (Pinto *et al.*, 2007*a*). Interestingly, the bismuth(III) salt showed a dual action, both as nucleophile donor and reaction promoter. Using this procedure, the ring opening of 5α,6α;16α,17α-diepoxy-20-oxopregnan-3β-yl acetate afforded regio-, stereo- and chemoselectively the title compound (I), bearing an intact 16α,17α-epoxide function (Pinto *et al.*, 2007*a*).

In order to unequivocally demonstrate the *trans*-diaxial nature of the ring opening of the 5α,6α;16α,17α-diepoxysteroid and the chemoselectivity for the epoxide fused to ring B, X-ray crystallography study was carried out on suitable single crystals of (I). Related X-ray diffraction studies on 5α-hydroxy-6β-substituted steroids have been recently published by our group (Pinto *et al.*, 2007*b*, 2008*b*, 2008*c*).

There are two symmetry independent molecules in the asymmetric unit (labeled A and B). No significant differences concerning bond lengths and angles were found between molecules A and B. All rings in both molecules are *trans*-fused. Ring A of molecules A and B adopts a chair conformation, although ring A of molecule B is more distorted, as shown by the Cremer and Pople (1975) puckering parameters [A: Q = 0.582 (3) Å, θ = 2.4 (4)°, φ = 233 (5)° B: Q = 0.583 (4) Å, θ = 6.1 (4)°, φ = 278 (4)°].

Rings B and C have a conformation close to chair. A C14-envelope conformation was found for the five-membered ring D of both molecules, with the following puckering parameters [A: q_2_ = 0.384 (3) Å and φ_2_ = 211.6 (5)°; B: q_2_ = 0.392 (3) Å and φ_2_ = 211.6 (5)°]. The acetoxy group at C3 and the methyl ketone side chain at C17 are both equatorial to ring A and D, respectively. The substituents at ring B are in axial positions. The hydroxyl at C5 is α-oriented while the bulky nitrate group show β-configuration. The epoxide group fused to the five-membered ring is below the plane of ring D, thus presenting a 16α,17α-configuration. The molecules are hydrogen-bonded *via* the 5α-hydroxyl and the C20 carbonyl groups acting as donor and acceptor, respectively.

### Experimental

The synthesis of 16α,17α-epoxy-5α-hydroxy-6β-nitrate-20-oxopregnan-3β-yl acetate (I) was efficiently accomplished by nucleophilic ring-opening of the corresponding 5α,6α;16α,17α-diepoxysteroid with bismuth(III) nitrate, in 1,4-dioxane (Pinto *et al.*, 2007*a*). The product of this reaction was isolated in 91% yield and identified as the title compound (I) from IR, ^1^H and ^13^C NMR spectroscopy data (Pinto *et al.*, 2007*a*). Recrystallization from methanol at room temperature gave colourless single crystals suitable for X-ray diffraction analysis.

### Refinement

All H atoms were refined as riding on their parent atoms using *SHELXL97* defaults, C—H ranging from 0.96 Å to 0.98 Å, O—H = 0.82 Å and U_iso_(H) set to 1.2U_eq_(C) or 1.5U_eq_(C_methyl_, O). In the absence of anomalous scatterers, Friedel pairs had been merged prior to refinement. The absolute configuration was not determined from the X-ray data but was known from the synthetic route.

### Crystal data

**Table:**

C_23_H_33_NO_8_	*Z* = 2
*M**_r_* = 451.50	*F*(000) = 484
Triclinic, *P*1	*D*_x_ = 1.288 Mg m^−^^3^
Hall symbol: P 1	Melting point: 455 K
*a* = 10.9740 (3) Å	Mo *K*α radiation, λ = 0.71073 Å
*b* = 11.0721 (3) Å	Cell parameters from 9093 reflections
*c* = 11.1686 (3) Å	θ = 2.3–28.3°
α = 77.2135 (15)°	µ = 0.10 mm^−^^1^
β = 73.2087 (14)°	*T* = 223 K
γ = 64.3465 (14)°	Truncated pyramid, clear colourless
*V* = 1163.81 (6) Å^3^	0.32 × 0.17 × 0.06 mm

### Data collection

**Table:**

Bruker APEXII CCD area-detector diffractometer	5770 independent reflections
Radiation source: fine-focus sealed tube	4806 reflections with *I* > 2σ(*I*)
graphite	*R*_int_ = 0.025
φ and ω scans	θ_max_ = 28.4°, θ_min_ = 1.9°
Absorption correction: multi-scan (*SADABS*; Sheldrick, 2000)	*h* = −14→14
*T*_min_ = 0.947, *T*_max_ = 0.994	*k* = −14→14
24934 measured reflections	*l* = −14→14

### Refinement

**Table:**

Refinement on *F*^2^	Primary atom site location: structure-invariant direct methods
Least-squares matrix: full	Secondary atom site location: difference Fourier map
*R*[*F*^2^ > 2σ(*F*^2^)] = 0.043	Hydrogen site location: inferred from neighbouring sites
*wR*(*F*^2^) = 0.118	H-atom parameters constrained
*S* = 1.04	*w* = 1/[σ^2^(*F*_o_^2^) + (0.0639*P*)^2^ + 0.1756*P*] where *P* = (*F*_o_^2^ + 2*F*_c_^2^)/3
5770 reflections	(Δ/σ)_max_ = 0.001
587 parameters	Δρ_max_ = 0.37 e Å^−^^3^
3 restraints	Δρ_min_ = −0.27 e Å^−^^3^

### Special details

**Table:**

Geometry. All e.s.d.'s (except the e.s.d. in the dihedral angle between two l.s. planes) are estimated using the full covariance matrix. The cell e.s.d.'s are taken into account individually in the estimation of e.s.d.'s in distances, angles and torsion angles; correlations between e.s.d.'s in cell parameters are only used when they are defined by crystal symmetry. An approximate (isotropic) treatment of cell e.s.d.'s is used for estimating e.s.d.'s involving l.s. planes.
Refinement. Refinement of *F*^2^ against ALL reflections. The weighted *R*-factor *wR* and goodness of fit *S* are based on *F*^2^, conventional *R*-factors *R* are based on *F*, with *F* set to zero for negative *F*^2^. The threshold expression of *F*^2^ > σ(*F*^2^) is used only for calculating *R*-factors(gt) *etc*. and is not relevant to the choice of reflections for refinement. *R*-factors based on *F*^2^ are statistically about twice as large as those based on *F*, and *R*- factors based on ALL data will be even larger.

### Fractional atomic coordinates and isotropic or equivalent isotropic displacement parameters (Å^2^)

**Table:**

	*x*	*y*	*z*	*U*_iso_*/*U*_eq_	
O3A	0.06020 (16)	0.62076 (15)	0.97526 (17)	0.0507 (4)	
O5A	0.25633 (16)	0.83696 (15)	0.69606 (17)	0.0485 (4)	
H5A	0.1910	0.8472	0.6676	0.073*	
O6A	0.48378 (19)	0.5008 (2)	0.6049 (2)	0.0654 (5)	
O7A	0.3044 (3)	0.4890 (4)	0.5533 (3)	0.1164 (12)	
O8A	0.4744 (3)	0.3104 (4)	0.6076 (4)	0.1458 (16)	
O16A	0.81145 (17)	0.95570 (17)	0.41953 (15)	0.0499 (4)	
O20A	1.02169 (17)	0.84865 (19)	0.62988 (16)	0.0544 (4)	
O22A	−0.1011 (2)	0.80189 (19)	1.0641 (2)	0.0823 (7)	
N6A	0.4134 (4)	0.4278 (4)	0.5858 (3)	0.0930 (11)	
C1A	0.3439 (2)	0.7488 (2)	0.9258 (2)	0.0432 (5)	
H1A1	0.4033	0.7384	0.9802	0.052*	
H1A2	0.2946	0.8446	0.9043	0.052*	
C2A	0.2381 (3)	0.6876 (3)	0.9985 (2)	0.0489 (5)	
H2A1	0.2866	0.5946	1.0301	0.059*	
H2A2	0.1797	0.7370	1.0700	0.059*	
C3A	0.1494 (2)	0.6932 (2)	0.9148 (2)	0.0425 (5)	
H3A	0.0919	0.7876	0.8918	0.051*	
C4A	0.2359 (2)	0.6276 (2)	0.7965 (2)	0.0420 (5)	
H4A1	0.1758	0.6352	0.7439	0.050*	
H4A2	0.2883	0.5326	0.8184	0.050*	
C5A	0.3353 (2)	0.6952 (2)	0.7239 (2)	0.0362 (4)	
C6A	0.4110 (2)	0.6457 (3)	0.5945 (2)	0.0489 (6)	
H6A	0.3419	0.6707	0.5444	0.059*	
C7A	0.5159 (3)	0.7067 (3)	0.5227 (2)	0.0492 (6)	
H7A1	0.4664	0.8002	0.4931	0.059*	
H7A2	0.5715	0.6593	0.4495	0.059*	
C8A	0.6127 (2)	0.7006 (2)	0.60137 (19)	0.0355 (4)	
H8A	0.6744	0.6066	0.6198	0.043*	
C9A	0.5271 (2)	0.7617 (2)	0.72606 (18)	0.0324 (4)	
H9A	0.4633	0.8532	0.7026	0.039*	
C10A	0.4350 (2)	0.6846 (2)	0.80441 (19)	0.0332 (4)	
C11A	0.6170 (2)	0.7770 (3)	0.8001 (2)	0.0428 (5)	
H11A	0.6731	0.6882	0.8348	0.051*	
H11B	0.5566	0.8281	0.8700	0.051*	
C12A	0.7133 (2)	0.8475 (2)	0.7209 (2)	0.0408 (5)	
H12A	0.6583	0.9412	0.6970	0.049*	
H12B	0.7739	0.8449	0.7707	0.049*	
C13A	0.8004 (2)	0.7773 (2)	0.60263 (18)	0.0335 (4)	
C14A	0.6984 (2)	0.7818 (2)	0.53019 (18)	0.0365 (4)	
H14A	0.6325	0.8759	0.5228	0.044*	
C15A	0.7847 (3)	0.7533 (3)	0.3968 (2)	0.0499 (6)	
H15A	0.8364	0.6574	0.3914	0.060*	
H15B	0.7269	0.7906	0.3355	0.060*	
C16A	0.8799 (3)	0.8247 (3)	0.3783 (2)	0.0504 (6)	
H16A	0.9628	0.8047	0.3097	0.060*	
C17A	0.8868 (2)	0.8440 (2)	0.5022 (2)	0.0385 (5)	
C18A	0.9024 (2)	0.6327 (2)	0.6365 (2)	0.0490 (5)	
H18A	0.9526	0.5897	0.5611	0.074*	
H18B	0.8517	0.5822	0.6918	0.074*	
H18C	0.9662	0.6362	0.6776	0.074*	
C19A	0.5259 (2)	0.5372 (2)	0.8420 (2)	0.0470 (5)	
H19A	0.5755	0.5341	0.9015	0.071*	
H19B	0.5906	0.4977	0.7686	0.071*	
H19C	0.4682	0.4877	0.8795	0.071*	
C20A	1.0047 (2)	0.8640 (2)	0.5232 (2)	0.0439 (5)	
C21A	1.0994 (3)	0.9042 (3)	0.4129 (3)	0.0666 (8)	
H21A	1.1512	0.9392	0.4412	0.100*	
H21B	1.0460	0.9724	0.3571	0.100*	
H21C	1.1620	0.8271	0.3693	0.100*	
C22A	−0.0654 (2)	0.6892 (2)	1.0404 (2)	0.0445 (5)	
C23A	−0.1511 (3)	0.6078 (3)	1.0851 (3)	0.0584 (7)	
H23A	−0.1086	0.5316	1.1416	0.088*	
H23B	−0.1580	0.5770	1.0144	0.088*	
H23C	−0.2420	0.6623	1.1282	0.088*	
O3B	0.03807 (17)	0.2481 (2)	0.42055 (18)	0.0665 (6)	
O5B	0.30588 (15)	0.31642 (16)	0.07748 (15)	0.0455 (4)	
H5B	0.2468	0.3132	0.0486	0.068*	
O6B	0.49821 (17)	−0.04860 (16)	0.14594 (16)	0.0485 (4)	
O7B	0.3139 (2)	−0.0808 (2)	0.13150 (19)	0.0636 (5)	
O8B	0.4702 (3)	−0.2329 (2)	0.2290 (2)	0.0815 (7)	
O16B	0.91153 (17)	0.29534 (16)	−0.21292 (14)	0.0464 (4)	
O20B	1.10024 (18)	0.26700 (19)	0.00177 (17)	0.0578 (5)	
O22B	−0.1340 (2)	0.4244 (3)	0.3654 (4)	0.1382 (16)	
N6B	0.4192 (3)	−0.1261 (2)	0.1694 (2)	0.0557 (6)	
C1B	0.3450 (2)	0.3480 (3)	0.3030 (2)	0.0499 (6)	
H1B1	0.3950	0.3674	0.3501	0.060*	
H1B2	0.3138	0.4252	0.2416	0.060*	
C2B	0.2177 (3)	0.3285 (3)	0.3940 (3)	0.0607 (7)	
H2B1	0.2476	0.2580	0.4612	0.073*	
H2B2	0.1563	0.4112	0.4314	0.073*	
C3B	0.1406 (2)	0.2911 (3)	0.3268 (2)	0.0526 (6)	
H3B	0.0941	0.3688	0.2707	0.063*	
C4B	0.2338 (2)	0.1719 (3)	0.2531 (2)	0.0437 (5)	
H4B1	0.1808	0.1562	0.2065	0.052*	
H4B2	0.2676	0.0919	0.3110	0.052*	
C5B	0.3569 (2)	0.1962 (2)	0.16149 (19)	0.0356 (4)	
C6B	0.4437 (2)	0.0847 (2)	0.0747 (2)	0.0403 (5)	
H6B	0.3854	0.0828	0.0238	0.048*	
C7B	0.5691 (2)	0.1061 (2)	−0.0130 (2)	0.0382 (5)	
H7B1	0.5377	0.1808	−0.0765	0.046*	
H7B2	0.6274	0.0261	−0.0557	0.046*	
C8B	0.6561 (2)	0.1351 (2)	0.05285 (19)	0.0319 (4)	
H8B	0.7023	0.0541	0.1061	0.038*	
C9B	0.5642 (2)	0.2522 (2)	0.13436 (18)	0.0324 (4)	
H9B	0.5205	0.3305	0.0772	0.039*	
C10B	0.4443 (2)	0.2234 (2)	0.23346 (19)	0.0361 (4)	
C11B	0.6523 (2)	0.2916 (2)	0.1920 (2)	0.0425 (5)	
H11C	0.5923	0.3710	0.2350	0.051*	
H11D	0.6914	0.2189	0.2543	0.051*	
C12B	0.7707 (2)	0.3208 (2)	0.0949 (2)	0.0398 (5)	
H12C	0.7322	0.4025	0.0404	0.048*	
H12D	0.8275	0.3351	0.1381	0.048*	
C13B	0.8598 (2)	0.2044 (2)	0.01604 (19)	0.0328 (4)	
C14B	0.7641 (2)	0.17770 (19)	−0.04354 (18)	0.0309 (4)	
H14B	0.7118	0.2649	−0.0859	0.037*	
C15B	0.8623 (2)	0.0906 (2)	−0.1492 (2)	0.0410 (5)	
H15C	0.9032	−0.0036	−0.1169	0.049*	
H15D	0.8152	0.0990	−0.2138	0.049*	
C16B	0.9698 (2)	0.1512 (2)	−0.1981 (2)	0.0446 (5)	
H16B	1.0586	0.1020	−0.2526	0.054*	
C17B	0.9659 (2)	0.2245 (2)	−0.1007 (2)	0.0391 (5)	
C18B	0.9412 (2)	0.0794 (2)	0.0968 (2)	0.0439 (5)	
H18D	0.9980	0.1012	0.1324	0.066*	
H18E	0.9989	0.0076	0.0451	0.066*	
H18F	0.8775	0.0514	0.1632	0.066*	
C19B	0.5032 (2)	0.1046 (3)	0.3303 (2)	0.0445 (5)	
H19D	0.5392	0.1321	0.3835	0.067*	
H19E	0.5762	0.0310	0.2873	0.067*	
H19F	0.4310	0.0763	0.3807	0.067*	
C20B	1.0865 (2)	0.2525 (3)	−0.0962 (2)	0.0491 (6)	
C21B	1.1848 (4)	0.2674 (5)	−0.2163 (3)	0.1020 (14)	
H21D	1.2457	0.3015	−0.2019	0.153*	
H21E	1.1339	0.3292	−0.2770	0.153*	
H21F	1.2383	0.1812	−0.2477	0.153*	
C22B	−0.0931 (2)	0.3207 (3)	0.4269 (2)	0.0508 (6)	
C23B	−0.1833 (3)	0.2606 (3)	0.5197 (3)	0.0667 (8)	
H23D	−0.2377	0.3183	0.5851	0.100*	
H23E	−0.1274	0.1739	0.5557	0.100*	
H23F	−0.2437	0.2504	0.4789	0.100*	

### Atomic displacement parameters (Å^2^)

**Table:**

	*U*^11^	*U*^22^	*U*^33^	*U*^12^	*U*^13^	*U*^23^
O3A	0.0356 (8)	0.0377 (8)	0.0753 (11)	−0.0206 (7)	0.0026 (8)	−0.0061 (7)
O5A	0.0339 (8)	0.0463 (8)	0.0693 (11)	−0.0195 (7)	−0.0230 (8)	0.0087 (8)
O6A	0.0541 (10)	0.0717 (12)	0.0871 (13)	−0.0415 (10)	0.0103 (9)	−0.0404 (10)
O7A	0.0903 (19)	0.188 (3)	0.126 (2)	−0.098 (2)	0.0199 (17)	−0.091 (2)
O8A	0.106 (2)	0.107 (2)	0.229 (4)	−0.0783 (19)	0.076 (2)	−0.101 (2)
O16A	0.0482 (9)	0.0594 (10)	0.0492 (9)	−0.0293 (8)	−0.0246 (8)	0.0158 (7)
O20A	0.0399 (9)	0.0807 (12)	0.0554 (10)	−0.0372 (9)	−0.0184 (8)	0.0065 (9)
O22A	0.0664 (12)	0.0499 (11)	0.1097 (17)	−0.0310 (10)	0.0361 (12)	−0.0248 (11)
N6A	0.080 (2)	0.112 (3)	0.108 (2)	−0.071 (2)	0.0487 (18)	−0.075 (2)
C1A	0.0394 (11)	0.0564 (13)	0.0418 (11)	−0.0281 (10)	−0.0023 (9)	−0.0106 (10)
C2A	0.0452 (13)	0.0540 (13)	0.0465 (13)	−0.0262 (11)	0.0040 (10)	−0.0083 (10)
C3A	0.0316 (10)	0.0338 (10)	0.0613 (14)	−0.0193 (9)	−0.0007 (10)	−0.0028 (10)
C4A	0.0317 (10)	0.0434 (11)	0.0574 (13)	−0.0194 (9)	−0.0130 (10)	−0.0042 (10)
C5A	0.0274 (9)	0.0408 (10)	0.0442 (11)	−0.0163 (8)	−0.0100 (8)	−0.0032 (9)
C6A	0.0429 (12)	0.0731 (16)	0.0485 (13)	−0.0348 (12)	−0.0119 (10)	−0.0133 (12)
C7A	0.0485 (13)	0.0788 (17)	0.0392 (12)	−0.0413 (13)	−0.0105 (10)	−0.0074 (11)
C8A	0.0317 (10)	0.0466 (11)	0.0345 (10)	−0.0202 (9)	−0.0083 (8)	−0.0048 (8)
C9A	0.0281 (9)	0.0409 (10)	0.0332 (9)	−0.0165 (8)	−0.0096 (8)	−0.0039 (8)
C10A	0.0279 (9)	0.0377 (10)	0.0375 (10)	−0.0161 (8)	−0.0086 (8)	−0.0027 (8)
C11A	0.0383 (11)	0.0677 (15)	0.0344 (11)	−0.0320 (11)	−0.0069 (9)	−0.0072 (10)
C12A	0.0357 (10)	0.0579 (13)	0.0419 (11)	−0.0284 (10)	−0.0110 (9)	−0.0063 (10)
C13A	0.0280 (9)	0.0407 (10)	0.0352 (10)	−0.0174 (8)	−0.0106 (8)	0.0020 (8)
C14A	0.0346 (10)	0.0501 (12)	0.0329 (10)	−0.0234 (9)	−0.0086 (8)	−0.0042 (9)
C15A	0.0549 (14)	0.0716 (16)	0.0355 (11)	−0.0386 (13)	−0.0080 (10)	−0.0032 (10)
C16A	0.0502 (13)	0.0709 (16)	0.0370 (11)	−0.0363 (13)	−0.0061 (10)	0.0023 (11)
C17A	0.0320 (10)	0.0434 (11)	0.0389 (11)	−0.0170 (9)	−0.0111 (8)	0.0066 (9)
C18A	0.0360 (11)	0.0484 (12)	0.0602 (14)	−0.0190 (10)	−0.0143 (11)	0.0086 (11)
C19A	0.0355 (11)	0.0468 (12)	0.0591 (14)	−0.0190 (10)	−0.0165 (10)	0.0070 (10)
C20A	0.0312 (10)	0.0485 (12)	0.0527 (13)	−0.0197 (9)	−0.0129 (10)	0.0061 (10)
C21A	0.0521 (15)	0.096 (2)	0.0631 (16)	−0.0508 (15)	−0.0147 (13)	0.0184 (15)
C22A	0.0390 (11)	0.0392 (11)	0.0514 (13)	−0.0207 (9)	0.0016 (10)	−0.0021 (9)
C23A	0.0462 (14)	0.0622 (15)	0.0719 (17)	−0.0346 (12)	0.0039 (12)	−0.0118 (13)
O3B	0.0297 (8)	0.0783 (12)	0.0635 (11)	−0.0161 (8)	0.0037 (8)	0.0164 (10)
O5B	0.0340 (8)	0.0600 (10)	0.0486 (9)	−0.0258 (7)	−0.0174 (7)	0.0090 (7)
O6B	0.0491 (9)	0.0516 (9)	0.0585 (10)	−0.0330 (8)	−0.0122 (8)	−0.0038 (7)
O7B	0.0630 (12)	0.0880 (14)	0.0672 (11)	−0.0559 (11)	−0.0044 (10)	−0.0193 (10)
O8B	0.1071 (19)	0.0662 (13)	0.0834 (15)	−0.0555 (13)	−0.0197 (13)	0.0114 (11)
O16B	0.0472 (9)	0.0549 (9)	0.0446 (8)	−0.0313 (8)	−0.0177 (7)	0.0133 (7)
O20B	0.0483 (10)	0.0788 (12)	0.0646 (11)	−0.0426 (9)	−0.0236 (9)	0.0085 (9)
O22B	0.0451 (13)	0.116 (2)	0.178 (3)	−0.0182 (13)	−0.0023 (16)	0.071 (2)
N6B	0.0648 (14)	0.0665 (14)	0.0500 (12)	−0.0458 (12)	0.0055 (11)	−0.0158 (11)
C1B	0.0364 (11)	0.0636 (15)	0.0512 (13)	−0.0185 (11)	−0.0018 (10)	−0.0224 (11)
C2B	0.0428 (13)	0.0787 (18)	0.0522 (14)	−0.0218 (13)	0.0102 (11)	−0.0233 (13)
C3B	0.0330 (11)	0.0666 (15)	0.0473 (13)	−0.0206 (11)	−0.0007 (10)	0.0059 (11)
C4B	0.0314 (10)	0.0632 (14)	0.0422 (11)	−0.0271 (10)	−0.0082 (9)	0.0007 (10)
C5B	0.0273 (9)	0.0504 (12)	0.0347 (10)	−0.0212 (9)	−0.0092 (8)	0.0000 (9)
C6B	0.0398 (11)	0.0520 (12)	0.0413 (11)	−0.0288 (10)	−0.0105 (9)	−0.0043 (9)
C7B	0.0360 (10)	0.0527 (12)	0.0370 (10)	−0.0283 (10)	−0.0019 (8)	−0.0117 (9)
C8B	0.0271 (9)	0.0371 (10)	0.0368 (10)	−0.0173 (8)	−0.0082 (8)	−0.0031 (8)
C9B	0.0263 (9)	0.0390 (10)	0.0361 (10)	−0.0152 (8)	−0.0087 (8)	−0.0050 (8)
C10B	0.0282 (9)	0.0486 (11)	0.0356 (10)	−0.0174 (9)	−0.0081 (8)	−0.0061 (9)
C11B	0.0372 (11)	0.0568 (13)	0.0448 (12)	−0.0242 (10)	−0.0086 (9)	−0.0159 (10)
C12B	0.0374 (11)	0.0429 (11)	0.0514 (12)	−0.0227 (9)	−0.0163 (10)	−0.0057 (9)
C13B	0.0277 (9)	0.0378 (10)	0.0383 (10)	−0.0179 (8)	−0.0126 (8)	0.0031 (8)
C14B	0.0268 (9)	0.0327 (9)	0.0362 (10)	−0.0151 (8)	−0.0083 (8)	−0.0010 (8)
C15B	0.0386 (11)	0.0469 (12)	0.0400 (11)	−0.0238 (10)	0.0000 (9)	−0.0066 (9)
C16B	0.0365 (11)	0.0535 (13)	0.0441 (12)	−0.0238 (10)	−0.0027 (9)	−0.0014 (10)
C17B	0.0314 (10)	0.0418 (11)	0.0447 (12)	−0.0192 (9)	−0.0128 (9)	0.0101 (9)
C18B	0.0362 (11)	0.0503 (12)	0.0474 (12)	−0.0231 (10)	−0.0168 (9)	0.0133 (10)
C19B	0.0372 (11)	0.0645 (14)	0.0362 (11)	−0.0248 (10)	−0.0110 (9)	−0.0004 (10)
C20B	0.0351 (11)	0.0604 (14)	0.0588 (14)	−0.0306 (11)	−0.0161 (11)	0.0121 (11)
C21B	0.081 (2)	0.190 (4)	0.073 (2)	−0.105 (3)	0.0000 (18)	−0.002 (2)
C22B	0.0357 (12)	0.0502 (13)	0.0587 (14)	−0.0153 (10)	−0.0060 (11)	−0.0006 (11)
C23B	0.0380 (13)	0.0806 (19)	0.0684 (18)	−0.0246 (13)	0.0039 (12)	−0.0014 (15)

### Geometric parameters (Å, °)

**Table:**

O3A—C22A	1.328 (3)	O3B—C22B	1.299 (3)
O3A—C3A	1.454 (2)	O3B—C3B	1.467 (3)
O5A—C5A	1.440 (3)	O5B—C5B	1.439 (3)
O5A—H5A	0.8200	O5B—H5B	0.8200
O6A—N6A	1.417 (3)	O6B—N6B	1.403 (2)
O6A—C6A	1.445 (3)	O6B—C6B	1.470 (3)
O7A—N6A	1.209 (5)	O7B—N6B	1.206 (3)
O8A—N6A	1.182 (5)	O8B—N6B	1.204 (3)
O16A—C16A	1.426 (3)	O16B—C16B	1.430 (3)
O16A—C17A	1.455 (2)	O16B—C17B	1.461 (2)
O20A—C20A	1.222 (3)	O20B—C20B	1.200 (3)
O22A—C22A	1.201 (3)	O22B—C22B	1.172 (3)
C1A—C2A	1.535 (3)	C1B—C2B	1.541 (3)
C1A—C10A	1.539 (3)	C1B—C10B	1.542 (3)
C1A—H1A1	0.9700	C1B—H1B1	0.9700
C1A—H1A2	0.9700	C1B—H1B2	0.9700
C2A—C3A	1.508 (3)	C2B—C3B	1.502 (4)
C2A—H2A1	0.9700	C2B—H2B1	0.9700
C2A—H2A2	0.9700	C2B—H2B2	0.9700
C3A—C4A	1.509 (3)	C3B—C4B	1.513 (4)
C3A—H3A	0.9800	C3B—H3B	0.9800
C4A—C5A	1.528 (3)	C4B—C5B	1.529 (3)
C4A—H4A1	0.9700	C4B—H4B1	0.9700
C4A—H4A2	0.9700	C4B—H4B2	0.9700
C5A—C6A	1.530 (3)	C5B—C6B	1.533 (3)
C5A—C10A	1.559 (3)	C5B—C10B	1.568 (3)
C6A—C7A	1.525 (3)	C6B—C7B	1.519 (3)
C6A—H6A	0.9800	C6B—H6B	0.9800
C7A—C8A	1.534 (3)	C7B—C8B	1.527 (3)
C7A—H7A1	0.9700	C7B—H7B1	0.9700
C7A—H7A2	0.9700	C7B—H7B2	0.9700
C8A—C14A	1.519 (3)	C8B—C14B	1.519 (3)
C8A—C9A	1.546 (3)	C8B—C9B	1.545 (3)
C8A—H8A	0.9800	C8B—H8B	0.9800
C9A—C11A	1.537 (3)	C9B—C11B	1.545 (3)
C9A—C10A	1.556 (3)	C9B—C10B	1.557 (3)
C9A—H9A	0.9800	C9B—H9B	0.9800
C10A—C19A	1.538 (3)	C10B—C19B	1.535 (3)
C11A—C12A	1.542 (3)	C11B—C12B	1.536 (3)
C11A—H11A	0.9700	C11B—H11C	0.9700
C11A—H11B	0.9700	C11B—H11D	0.9700
C12A—C13A	1.531 (3)	C12B—C13B	1.519 (3)
C12A—H12A	0.9700	C12B—H12C	0.9700
C12A—H12B	0.9700	C12B—H12D	0.9700
C13A—C17A	1.525 (3)	C13B—C17B	1.525 (3)
C13A—C14A	1.539 (3)	C13B—C18B	1.540 (3)
C13A—C18A	1.544 (3)	C13B—C14B	1.547 (3)
C14A—C15A	1.533 (3)	C14B—C15B	1.534 (3)
C14A—H14A	0.9800	C14B—H14B	0.9800
C15A—C16A	1.509 (3)	C15B—C16B	1.514 (3)
C15A—H15A	0.9700	C15B—H15C	0.9700
C15A—H15B	0.9700	C15B—H15D	0.9700
C16A—C17A	1.474 (3)	C16B—C17B	1.476 (3)
C16A—H16A	0.9800	C16B—H16B	0.9800
C17A—C20A	1.491 (3)	C17B—C20B	1.501 (3)
C18A—H18A	0.9600	C18B—H18D	0.9600
C18A—H18B	0.9600	C18B—H18E	0.9600
C18A—H18C	0.9600	C18B—H18F	0.9600
C19A—H19A	0.9600	C19B—H19D	0.9600
C19A—H19B	0.9600	C19B—H19E	0.9600
C19A—H19C	0.9600	C19B—H19F	0.9600
C20A—C21A	1.488 (3)	C20B—C21B	1.487 (4)
C21A—H21A	0.9600	C21B—H21D	0.9600
C21A—H21B	0.9600	C21B—H21E	0.9600
C21A—H21C	0.9600	C21B—H21F	0.9600
C22A—C23A	1.486 (3)	C22B—C23B	1.473 (4)
C23A—H23A	0.9600	C23B—H23D	0.9600
C23A—H23B	0.9600	C23B—H23E	0.9600
C23A—H23C	0.9600	C23B—H23F	0.9600
			
C22A—O3A—C3A	117.75 (16)	C22B—O3B—C3B	119.48 (19)
C5A—O5A—H5A	109.5	C5B—O5B—H5B	109.5
N6A—O6A—C6A	115.8 (2)	N6B—O6B—C6B	115.81 (17)
C16A—O16A—C17A	61.54 (15)	C16B—O16B—C17B	61.39 (14)
O8A—N6A—O7A	129.8 (3)	O8B—N6B—O7B	129.0 (2)
O8A—N6A—O6A	111.1 (4)	O8B—N6B—O6B	111.6 (2)
O7A—N6A—O6A	119.1 (3)	O7B—N6B—O6B	119.4 (2)
C2A—C1A—C10A	113.77 (18)	C2B—C1B—C10B	112.8 (2)
C2A—C1A—H1A1	108.8	C2B—C1B—H1B1	109.0
C10A—C1A—H1A1	108.8	C10B—C1B—H1B1	109.0
C2A—C1A—H1A2	108.8	C2B—C1B—H1B2	109.0
C10A—C1A—H1A2	108.8	C10B—C1B—H1B2	109.0
H1A1—C1A—H1A2	107.7	H1B1—C1B—H1B2	107.8
C3A—C2A—C1A	110.73 (19)	C3B—C2B—C1B	111.1 (2)
C3A—C2A—H2A1	109.5	C3B—C2B—H2B1	109.4
C1A—C2A—H2A1	109.5	C1B—C2B—H2B1	109.4
C3A—C2A—H2A2	109.5	C3B—C2B—H2B2	109.4
C1A—C2A—H2A2	109.5	C1B—C2B—H2B2	109.4
H2A1—C2A—H2A2	108.1	H2B1—C2B—H2B2	108.0
O3A—C3A—C2A	112.36 (19)	O3B—C3B—C2B	108.8 (2)
O3A—C3A—C4A	105.21 (17)	O3B—C3B—C4B	105.5 (2)
C2A—C3A—C4A	111.78 (18)	C2B—C3B—C4B	112.6 (2)
O3A—C3A—H3A	109.1	O3B—C3B—H3B	109.9
C2A—C3A—H3A	109.1	C2B—C3B—H3B	109.9
C4A—C3A—H3A	109.1	C4B—C3B—H3B	109.9
C3A—C4A—C5A	110.32 (17)	C3B—C4B—C5B	111.33 (19)
C3A—C4A—H4A1	109.6	C3B—C4B—H4B1	109.4
C5A—C4A—H4A1	109.6	C5B—C4B—H4B1	109.4
C3A—C4A—H4A2	109.6	C3B—C4B—H4B2	109.4
C5A—C4A—H4A2	109.6	C5B—C4B—H4B2	109.4
H4A1—C4A—H4A2	108.1	H4B1—C4B—H4B2	108.0
O5A—C5A—C4A	108.95 (16)	O5B—C5B—C4B	109.03 (16)
O5A—C5A—C6A	104.13 (18)	O5B—C5B—C6B	104.41 (16)
C4A—C5A—C6A	112.31 (18)	C4B—C5B—C6B	112.52 (17)
O5A—C5A—C10A	106.21 (16)	O5B—C5B—C10B	106.11 (16)
C4A—C5A—C10A	111.51 (17)	C4B—C5B—C10B	110.82 (16)
C6A—C5A—C10A	113.19 (16)	C6B—C5B—C10B	113.46 (16)
O6A—C6A—C7A	107.7 (2)	O6B—C6B—C7B	105.97 (18)
O6A—C6A—C5A	111.27 (19)	O6B—C6B—C5B	111.95 (17)
C7A—C6A—C5A	113.30 (18)	C7B—C6B—C5B	112.54 (17)
O6A—C6A—H6A	108.1	O6B—C6B—H6B	108.8
C7A—C6A—H6A	108.1	C7B—C6B—H6B	108.8
C5A—C6A—H6A	108.1	C5B—C6B—H6B	108.8
C6A—C7A—C8A	113.66 (18)	C6B—C7B—C8B	114.21 (17)
C6A—C7A—H7A1	108.8	C6B—C7B—H7B1	108.7
C8A—C7A—H7A1	108.8	C8B—C7B—H7B1	108.7
C6A—C7A—H7A2	108.8	C6B—C7B—H7B2	108.7
C8A—C7A—H7A2	108.8	C8B—C7B—H7B2	108.7
H7A1—C7A—H7A2	107.7	H7B1—C7B—H7B2	107.6
C14A—C8A—C7A	110.26 (17)	C14B—C8B—C7B	110.35 (16)
C14A—C8A—C9A	107.88 (17)	C14B—C8B—C9B	107.18 (15)
C7A—C8A—C9A	110.20 (17)	C7B—C8B—C9B	110.61 (15)
C14A—C8A—H8A	109.5	C14B—C8B—H8B	109.6
C7A—C8A—H8A	109.5	C7B—C8B—H8B	109.6
C9A—C8A—H8A	109.5	C9B—C8B—H8B	109.6
C11A—C9A—C8A	112.49 (16)	C8B—C9B—C11B	111.29 (16)
C11A—C9A—C10A	113.98 (16)	C8B—C9B—C10B	112.15 (16)
C8A—C9A—C10A	111.10 (16)	C11B—C9B—C10B	113.40 (16)
C11A—C9A—H9A	106.2	C8B—C9B—H9B	106.5
C8A—C9A—H9A	106.2	C11B—C9B—H9B	106.5
C10A—C9A—H9A	106.2	C10B—C9B—H9B	106.5
C19A—C10A—C1A	107.93 (18)	C19B—C10B—C1B	108.82 (19)
C19A—C10A—C9A	110.37 (16)	C19B—C10B—C9B	110.16 (16)
C1A—C10A—C9A	111.64 (16)	C1B—C10B—C9B	110.93 (17)
C19A—C10A—C5A	111.83 (17)	C19B—C10B—C5B	112.43 (18)
C1A—C10A—C5A	107.02 (16)	C1B—C10B—C5B	106.45 (16)
C9A—C10A—C5A	108.04 (15)	C9B—C10B—C5B	108.00 (15)
C9A—C11A—C12A	114.04 (17)	C12B—C11B—C9B	113.84 (17)
C9A—C11A—H11A	108.7	C12B—C11B—H11C	108.8
C12A—C11A—H11A	108.7	C9B—C11B—H11C	108.8
C9A—C11A—H11B	108.7	C12B—C11B—H11D	108.8
C12A—C11A—H11B	108.7	C9B—C11B—H11D	108.8
H11A—C11A—H11B	107.6	H11C—C11B—H11D	107.7
C13A—C12A—C11A	110.20 (17)	C13B—C12B—C11B	110.84 (16)
C13A—C12A—H12A	109.6	C13B—C12B—H12C	109.5
C11A—C12A—H12A	109.6	C11B—C12B—H12C	109.5
C13A—C12A—H12B	109.6	C13B—C12B—H12D	109.5
C11A—C12A—H12B	109.6	C11B—C12B—H12D	109.5
H12A—C12A—H12B	108.1	H12C—C12B—H12D	108.1
C17A—C13A—C12A	118.28 (17)	C12B—C13B—C17B	118.65 (17)
C17A—C13A—C14A	101.29 (15)	C12B—C13B—C18B	110.49 (18)
C12A—C13A—C14A	106.94 (16)	C17B—C13B—C18B	105.91 (16)
C17A—C13A—C18A	105.86 (16)	C12B—C13B—C14B	108.06 (16)
C12A—C13A—C18A	111.21 (17)	C17B—C13B—C14B	100.91 (16)
C14A—C13A—C18A	113.05 (18)	C18B—C13B—C14B	112.58 (16)
C8A—C14A—C15A	120.13 (18)	C8B—C14B—C15B	120.56 (16)
C8A—C14A—C13A	113.00 (16)	C8B—C14B—C13B	113.40 (16)
C15A—C14A—C13A	104.83 (17)	C15B—C14B—C13B	104.89 (16)
C8A—C14A—H14A	106.0	C8B—C14B—H14B	105.6
C15A—C14A—H14A	106.0	C15B—C14B—H14B	105.6
C13A—C14A—H14A	106.0	C13B—C14B—H14B	105.6
C16A—C15A—C14A	101.89 (18)	C16B—C15B—C14B	101.39 (17)
C16A—C15A—H15A	111.4	C16B—C15B—H15C	111.5
C14A—C15A—H15A	111.4	C14B—C15B—H15C	111.5
C16A—C15A—H15B	111.4	C16B—C15B—H15D	111.5
C14A—C15A—H15B	111.4	C14B—C15B—H15D	111.5
H15A—C15A—H15B	109.3	H15C—C15B—H15D	109.3
O16A—C16A—C17A	60.22 (14)	O16B—C16B—C17B	60.32 (14)
O16A—C16A—C15A	113.6 (2)	O16B—C16B—C15B	113.52 (18)
C17A—C16A—C15A	109.16 (19)	C17B—C16B—C15B	109.40 (18)
O16A—C16A—H16A	119.8	O16B—C16B—H16B	119.8
C17A—C16A—H16A	119.8	C17B—C16B—H16B	119.8
C15A—C16A—H16A	119.8	C15B—C16B—H16B	119.8
O16A—C17A—C16A	58.24 (15)	O16B—C17B—C16B	58.29 (14)
O16A—C17A—C20A	111.10 (17)	O16B—C17B—C20B	111.54 (17)
C16A—C17A—C20A	123.0 (2)	C16B—C17B—C20B	123.1 (2)
O16A—C17A—C13A	115.75 (16)	O16B—C17B—C13B	115.73 (16)
C16A—C17A—C13A	107.69 (18)	C16B—C17B—C13B	107.64 (17)
C20A—C17A—C13A	123.65 (17)	C20B—C17B—C13B	123.40 (19)
C13A—C18A—H18A	109.5	C13B—C18B—H18D	109.5
C13A—C18A—H18B	109.5	C13B—C18B—H18E	109.5
H18A—C18A—H18B	109.5	H18D—C18B—H18E	109.5
C13A—C18A—H18C	109.5	C13B—C18B—H18F	109.5
H18A—C18A—H18C	109.5	H18D—C18B—H18F	109.5
H18B—C18A—H18C	109.5	H18E—C18B—H18F	109.5
C10A—C19A—H19A	109.5	C10B—C19B—H19D	109.5
C10A—C19A—H19B	109.5	C10B—C19B—H19E	109.5
H19A—C19A—H19B	109.5	H19D—C19B—H19E	109.5
C10A—C19A—H19C	109.5	C10B—C19B—H19F	109.5
H19A—C19A—H19C	109.5	H19D—C19B—H19F	109.5
H19B—C19A—H19C	109.5	H19E—C19B—H19F	109.5
O20A—C20A—C21A	120.9 (2)	O20B—C20B—C21B	121.0 (2)
O20A—C20A—C17A	120.1 (2)	O20B—C20B—C17B	120.5 (2)
C21A—C20A—C17A	119.0 (2)	C21B—C20B—C17B	118.4 (2)
C20A—C21A—H21A	109.5	C20B—C21B—H21D	109.5
C20A—C21A—H21B	109.5	C20B—C21B—H21E	109.5
H21A—C21A—H21B	109.5	H21D—C21B—H21E	109.5
C20A—C21A—H21C	109.5	C20B—C21B—H21F	109.5
H21A—C21A—H21C	109.5	H21D—C21B—H21F	109.5
H21B—C21A—H21C	109.5	H21E—C21B—H21F	109.5
O22A—C22A—O3A	122.7 (2)	O22B—C22B—O3B	122.6 (2)
O22A—C22A—C23A	124.9 (2)	O22B—C22B—C23B	124.0 (2)
O3A—C22A—C23A	112.3 (2)	O3B—C22B—C23B	113.3 (2)
C22A—C23A—H23A	109.5	C22B—C23B—H23D	109.5
C22A—C23A—H23B	109.5	C22B—C23B—H23E	109.5
H23A—C23A—H23B	109.5	H23D—C23B—H23E	109.5
C22A—C23A—H23C	109.5	C22B—C23B—H23F	109.5
H23A—C23A—H23C	109.5	H23D—C23B—H23F	109.5
H23B—C23A—H23C	109.5	H23E—C23B—H23F	109.5
			
C6A—O6A—N6A—O8A	−173.4 (3)	C6B—O6B—N6B—O8B	−179.4 (2)
C6A—O6A—N6A—O7A	5.4 (4)	C6B—O6B—N6B—O7B	−0.2 (3)
C10A—C1A—C2A—C3A	−54.8 (3)	C10B—C1B—C2B—C3B	−55.8 (3)
C22A—O3A—C3A—C2A	90.6 (2)	C22B—O3B—C3B—C2B	113.4 (3)
C22A—O3A—C3A—C4A	−147.5 (2)	C22B—O3B—C3B—C4B	−125.5 (3)
C1A—C2A—C3A—O3A	172.55 (18)	C1B—C2B—C3B—O3B	169.0 (2)
C1A—C2A—C3A—C4A	54.5 (2)	C1B—C2B—C3B—C4B	52.4 (3)
O3A—C3A—C4A—C5A	−179.87 (17)	O3B—C3B—C4B—C5B	−173.26 (17)
C2A—C3A—C4A—C5A	−57.7 (2)	C2B—C3B—C4B—C5B	−54.7 (3)
C3A—C4A—C5A—O5A	−57.2 (2)	C3B—C4B—C5B—O5B	−58.0 (2)
C3A—C4A—C5A—C6A	−172.08 (18)	C3B—C4B—C5B—C6B	−173.30 (18)
C3A—C4A—C5A—C10A	59.7 (2)	C3B—C4B—C5B—C10B	58.5 (2)
N6A—O6A—C6A—C7A	−138.5 (2)	N6B—O6B—C6B—C7B	−141.69 (18)
N6A—O6A—C6A—C5A	96.8 (2)	N6B—O6B—C6B—C5B	95.3 (2)
O5A—C5A—C6A—O6A	−173.49 (17)	O5B—C5B—C6B—O6B	−176.68 (15)
C4A—C5A—C6A—O6A	−55.8 (2)	C4B—C5B—C6B—O6B	−58.6 (2)
C10A—C5A—C6A—O6A	71.6 (2)	C10B—C5B—C6B—O6B	68.2 (2)
O5A—C5A—C6A—C7A	65.0 (2)	O5B—C5B—C6B—C7B	64.1 (2)
C4A—C5A—C6A—C7A	−177.25 (19)	C4B—C5B—C6B—C7B	−177.82 (18)
C10A—C5A—C6A—C7A	−49.9 (3)	C10B—C5B—C6B—C7B	−51.0 (2)
O6A—C6A—C7A—C8A	−75.3 (2)	O6B—C6B—C7B—C8B	−73.1 (2)
C5A—C6A—C7A—C8A	48.2 (3)	C5B—C6B—C7B—C8B	49.5 (2)
C6A—C7A—C8A—C14A	−171.4 (2)	C6B—C7B—C8B—C14B	−170.62 (17)
C6A—C7A—C8A—C9A	−52.4 (3)	C6B—C7B—C8B—C9B	−52.2 (2)
C14A—C8A—C9A—C11A	−51.3 (2)	C14B—C8B—C9B—C11B	−54.5 (2)
C7A—C8A—C9A—C11A	−171.77 (19)	C7B—C8B—C9B—C11B	−174.82 (17)
C14A—C8A—C9A—C10A	179.52 (16)	C14B—C8B—C9B—C10B	177.30 (15)
C7A—C8A—C9A—C10A	59.1 (2)	C7B—C8B—C9B—C10B	57.0 (2)
C2A—C1A—C10A—C19A	−65.6 (2)	C2B—C1B—C10B—C19B	−63.6 (3)
C2A—C1A—C10A—C9A	172.89 (18)	C2B—C1B—C10B—C9B	175.0 (2)
C2A—C1A—C10A—C5A	54.9 (2)	C2B—C1B—C10B—C5B	57.8 (3)
C11A—C9A—C10A—C19A	−65.5 (2)	C8B—C9B—C10B—C19B	65.8 (2)
C8A—C9A—C10A—C19A	62.8 (2)	C11B—C9B—C10B—C19B	−61.3 (2)
C11A—C9A—C10A—C1A	54.5 (2)	C8B—C9B—C10B—C1B	−173.61 (17)
C8A—C9A—C10A—C1A	−177.16 (16)	C11B—C9B—C10B—C1B	59.3 (2)
C11A—C9A—C10A—C5A	171.91 (17)	C8B—C9B—C10B—C5B	−57.3 (2)
C8A—C9A—C10A—C5A	−59.7 (2)	C11B—C9B—C10B—C5B	175.60 (17)
O5A—C5A—C10A—C19A	179.61 (18)	O5B—C5B—C10B—C19B	178.45 (17)
C4A—C5A—C10A—C19A	61.0 (2)	C4B—C5B—C10B—C19B	60.2 (2)
C6A—C5A—C10A—C19A	−66.7 (2)	C6B—C5B—C10B—C19B	−67.5 (2)
O5A—C5A—C10A—C1A	61.6 (2)	O5B—C5B—C10B—C1B	59.4 (2)
C4A—C5A—C10A—C1A	−56.9 (2)	C4B—C5B—C10B—C1B	−58.8 (2)
C6A—C5A—C10A—C1A	175.27 (19)	C6B—C5B—C10B—C1B	173.45 (18)
O5A—C5A—C10A—C9A	−58.7 (2)	O5B—C5B—C10B—C9B	−59.8 (2)
C4A—C5A—C10A—C9A	−177.30 (16)	C4B—C5B—C10B—C9B	−178.03 (17)
C6A—C5A—C10A—C9A	54.9 (2)	C6B—C5B—C10B—C9B	54.3 (2)
C8A—C9A—C11A—C12A	49.7 (3)	C8B—C9B—C11B—C12B	52.7 (2)
C10A—C9A—C11A—C12A	177.32 (18)	C10B—C9B—C11B—C12B	−179.77 (17)
C9A—C11A—C12A—C13A	−53.0 (2)	C9B—C11B—C12B—C13B	−53.0 (2)
C11A—C12A—C13A—C17A	171.14 (17)	C11B—C12B—C13B—C17B	168.92 (17)
C11A—C12A—C13A—C14A	57.8 (2)	C11B—C12B—C13B—C18B	−68.6 (2)
C11A—C12A—C13A—C18A	−66.1 (2)	C11B—C12B—C13B—C14B	55.0 (2)
C7A—C8A—C14A—C15A	−54.1 (3)	C7B—C8B—C14B—C15B	−52.6 (2)
C9A—C8A—C14A—C15A	−174.47 (19)	C9B—C8B—C14B—C15B	−173.11 (17)
C7A—C8A—C14A—C13A	−178.69 (19)	C7B—C8B—C14B—C13B	−178.11 (17)
C9A—C8A—C14A—C13A	60.9 (2)	C9B—C8B—C14B—C13B	61.38 (19)
C17A—C13A—C14A—C8A	170.59 (17)	C12B—C13B—C14B—C8B	−62.4 (2)
C12A—C13A—C14A—C8A	−65.0 (2)	C17B—C13B—C14B—C8B	172.37 (16)
C18A—C13A—C14A—C8A	57.8 (2)	C18B—C13B—C14B—C8B	59.9 (2)
C17A—C13A—C14A—C15A	38.0 (2)	C12B—C13B—C14B—C15B	164.07 (16)
C12A—C13A—C14A—C15A	162.47 (18)	C17B—C13B—C14B—C15B	38.87 (19)
C18A—C13A—C14A—C15A	−74.8 (2)	C18B—C13B—C14B—C15B	−73.6 (2)
C8A—C14A—C15A—C16A	−164.78 (19)	C8B—C14B—C15B—C16B	−166.35 (17)
C13A—C14A—C15A—C16A	−36.4 (2)	C13B—C14B—C15B—C16B	−37.0 (2)
C17A—O16A—C16A—C15A	99.3 (2)	C17B—O16B—C16B—C15B	99.7 (2)
C14A—C15A—C16A—O16A	−44.4 (2)	C14B—C15B—C16B—O16B	−44.3 (2)
C14A—C15A—C16A—C17A	20.6 (3)	C14B—C15B—C16B—C17B	20.9 (2)
C16A—O16A—C17A—C20A	116.6 (2)	C16B—O16B—C17B—C20B	116.5 (2)
C16A—O16A—C17A—C13A	−95.6 (2)	C16B—O16B—C17B—C13B	−95.6 (2)
C15A—C16A—C17A—O16A	−106.9 (2)	C15B—C16B—C17B—O16B	−106.63 (19)
O16A—C16A—C17A—C20A	−95.9 (2)	O16B—C16B—C17B—C20B	−96.5 (2)
C15A—C16A—C17A—C20A	157.2 (2)	C15B—C16B—C17B—C20B	156.9 (2)
O16A—C16A—C17A—C13A	109.81 (17)	O16B—C16B—C17B—C13B	109.81 (17)
C15A—C16A—C17A—C13A	2.9 (3)	C15B—C16B—C17B—C13B	3.2 (2)
C12A—C13A—C17A—O16A	−78.8 (2)	C12B—C13B—C17B—O16B	−80.6 (2)
C14A—C13A—C17A—O16A	37.6 (2)	C18B—C13B—C17B—O16B	154.57 (18)
C18A—C13A—C17A—O16A	155.71 (19)	C14B—C13B—C17B—O16B	37.1 (2)
C12A—C13A—C17A—C16A	−141.5 (2)	C12B—C13B—C17B—C16B	−143.32 (19)
C14A—C13A—C17A—C16A	−25.1 (2)	C18B—C13B—C17B—C16B	91.89 (19)
C18A—C13A—C17A—C16A	93.1 (2)	C14B—C13B—C17B—C16B	−25.6 (2)
C12A—C13A—C17A—C20A	64.4 (3)	C12B—C13B—C17B—C20B	63.1 (3)
C14A—C13A—C17A—C20A	−179.2 (2)	C18B—C13B—C17B—C20B	−61.7 (3)
C18A—C13A—C17A—C20A	−61.0 (3)	C14B—C13B—C17B—C20B	−179.23 (19)
O16A—C17A—C20A—O20A	131.8 (2)	O16B—C17B—C20B—O20B	141.3 (2)
C16A—C17A—C20A—O20A	−163.2 (2)	C16B—C17B—C20B—O20B	−153.3 (2)
C13A—C17A—C20A—O20A	−12.9 (3)	C13B—C17B—C20B—O20B	−3.7 (4)
O16A—C17A—C20A—C21A	−47.5 (3)	O16B—C17B—C20B—C21B	−36.2 (4)
C16A—C17A—C20A—C21A	17.5 (3)	C16B—C17B—C20B—C21B	29.1 (4)
C13A—C17A—C20A—C21A	167.8 (2)	C13B—C17B—C20B—C21B	178.8 (3)
C3A—O3A—C22A—O22A	−9.5 (4)	C3B—O3B—C22B—O22B	−4.5 (5)
C3A—O3A—C22A—C23A	173.6 (2)	C3B—O3B—C22B—C23B	176.3 (2)

### Hydrogen-bond geometry (Å, °)

**Table:**

*D*—H···*A*	*D*—H	H···*A*	*D*···*A*	*D*—H···*A*
O5A—H5A···O20A^i^	0.82	2.01	2.822 (2)	168
O5B—H5B···O20B^i^	0.82	2.11	2.913 (2)	168

Symmetry codes: (i) *x*−1, *y*, *z*.
